# Miniaturised Low-Cost Gamma Scanning Platform for Contamination Identification, Localisation and Characterisation: A New Instrument in the Decommissioning Toolkit

**DOI:** 10.3390/s21082884

**Published:** 2021-04-20

**Authors:** Yannick Verbelen, Peter G. Martin, Kamran Ahmad, Suresh Kaluvan, Thomas B. Scott

**Affiliations:** 1 Interface Analysis Centre, School of Physics, University of Bristol, Bristol BS8 1TL, UK; peter.martin@bristol.ac.uk (P.G.M.); suresh.kaluvan@bristol.ac.uk (S.K.); t.b.scott@bristol.ac.uk (T.B.S.); 2 ImiTec Ltd., Unit 14 Greenway Farm, Bristol BS30 5RL, UK; kamran.ahmad@imitec.co.uk

**Keywords:** gamma spectrometry, nuclear decommissioning, point cloud data, cell characterisation, sensor fusion

## Abstract

Formerly clandestine, abandoned and legacy nuclear facilities, whether associated with civil or military applications, represent a significant decommissioning challenge owing to the lack of knowledge surrounding the existence, location and types of radioactive material(s) that may be present. Consequently, mobile and highly deployable systems that are able to identify, spatially locate and compositionally assay contamination ahead of remedial actions are of vital importance. Deployment imposes constraints to dimensions resulting from small diameter access ports or pipes. Herein, we describe a prototype low-cost, miniaturised and rapidly deployable ‘cell characterisation’ gamma-ray scanning system to allow for the examination of enclosed (internal) or outdoor (external) spaces for radioactive ‘hot-spots’. The readout from the miniaturised and lead-collimated gamma-ray spectrometer, that is progressively rastered through a stepped snake motion, is combined with distance measurements derived from a single-point laser range-finder to obtain an array of measurements in order to yield a 3-dimensional point-cloud, based on a polar coordinate system—scaled for radiation intensity. Existing as a smaller and more cost-effective platform than presently available, we are able to produce a millimetre-accurate 3D volumetric rendering of a space—whether internal or external, onto which fully spectroscopic radiation intensity data can be overlain to pinpoint the exact positions at which (even low abundance) gamma-emitting materials exist.

## 1. Introduction

Resulting from the increasing global drive toward the restoration and remediation of legacy nuclear facilities, technology to aid in such tasks is becoming more developed and widely utilised. Of particular interest are small and readily deployable systems that can be rapidly exploited to locate and fingerprint radioactive materials across a range of radiologically contaminated environments. The ideal system should ideally possess the following attributes:

1. A detector capability to coincidentally record distance to a point on a surface and the radiation energy and intensity being emitted from that position.
2. Ability to pan and tilt the detector to analyse the entire volume of the surrounding area in order to create a hybrid positional-radiation point cloud.
3. Ability to operate autonomously and be controlled/programmed remotely; such that human operators can remain at a safe distance from any radioactive environments.
4. Is sufficiently compact and light-weight, such that it can easily be deployed on a mobile robotic platform or manipulator.

The historic and established approach to identifying isolated regions of radioactive contamination utilise pinhole cameras, first proposed by L. L. Wiltshire in 1961 for military purposes [1]. The idea was expanded by Greenfield et al. to formalize the design constraints of a pinhole camera [2]. CEA LIST subsequently developed the concept and adapted it to build the first generation gamma radiation camera, the Cartogram [3], since being commercialised by Areva Canberra. Although it proved flexible, its weight of 15 kg turned out to be a limiting factor in decommissioning applications where weight is of concern. Pinhole designs continue to be improved to date, with recent advancements for high dose rate medical imaging developed by Yamamoto et al. [4]. An alternative approach to the pinhole collimator concept uses coded masks, which saves weight and improves accuracy [3]. This principle, first developed for medical imaging using a 1 mm thick CdTe detector substrate (Medipix2), was improved further in the Gampix gamma camera [5]. Usage of semiconductor detectors and silicon photomultipliers (SiPMs) enables further weight reductions in comparison to legacy photomultiplier tubes (PMTs) [6], although both approaches are the subject of current research efforts. With a focus on weight and size reduction, Compton cameras are drawing increased attention from the scientific community because heavy and bulky collimators can be omitted [7], resulting in compact systems such as the *Nanopix* [8]. Gamma cameras can also be constructed with commercial detectors, such as the Kromek Sigma and GR1 [9] (p. 5, Figure 2), searching for a trade-off between accuracy, handling, and ease of deployment. Recent work by Liu et al. has advanced the image reconstruction algorithms for scintillator-based gamma cameras such as the Kromek Sigma [10], although the presented solutions can also be applied to semiconductor based cameras. Yuki Sato and Tatsuo Torii offer a comprehensive solution for a gamma camera, overlaying radiation data with point cloud data (PCD) obtained with LiDAR and VIS cameras [9,11], with as significant drawback the size of their experimental setup as well as their cost, and a limited field of view (FOV). Particularly in nuclear facilities with high radiation dose rates, such as nuclear power plants or nuclear reprocessing facilities, access to areas is often restricted to small diameter ports with a diameter of ca. 20–30 cm. A gamma camera therefore either needs to be very compact, or able to unfold itself. In these highly active cells, size and autonomy are more critical factors than accuracy or the time needed to complete a scan, therefore we have produced the ‘Cell Characterisation-Remote Isotopic Analysis System (CC-RIAS). The ‘CC-RIAS’ proposes a solution for the limited FOV by combining a compact directional semiconductor radiation detector with a pan-tilt-unit (PTU). Its aim is to offer a compact, low-cost (disposable if necessary) gamma camera capable of generating a PCD in nuclear decommissioning environments.

## 2. Instrument Design and Setup

**Figure 1:**
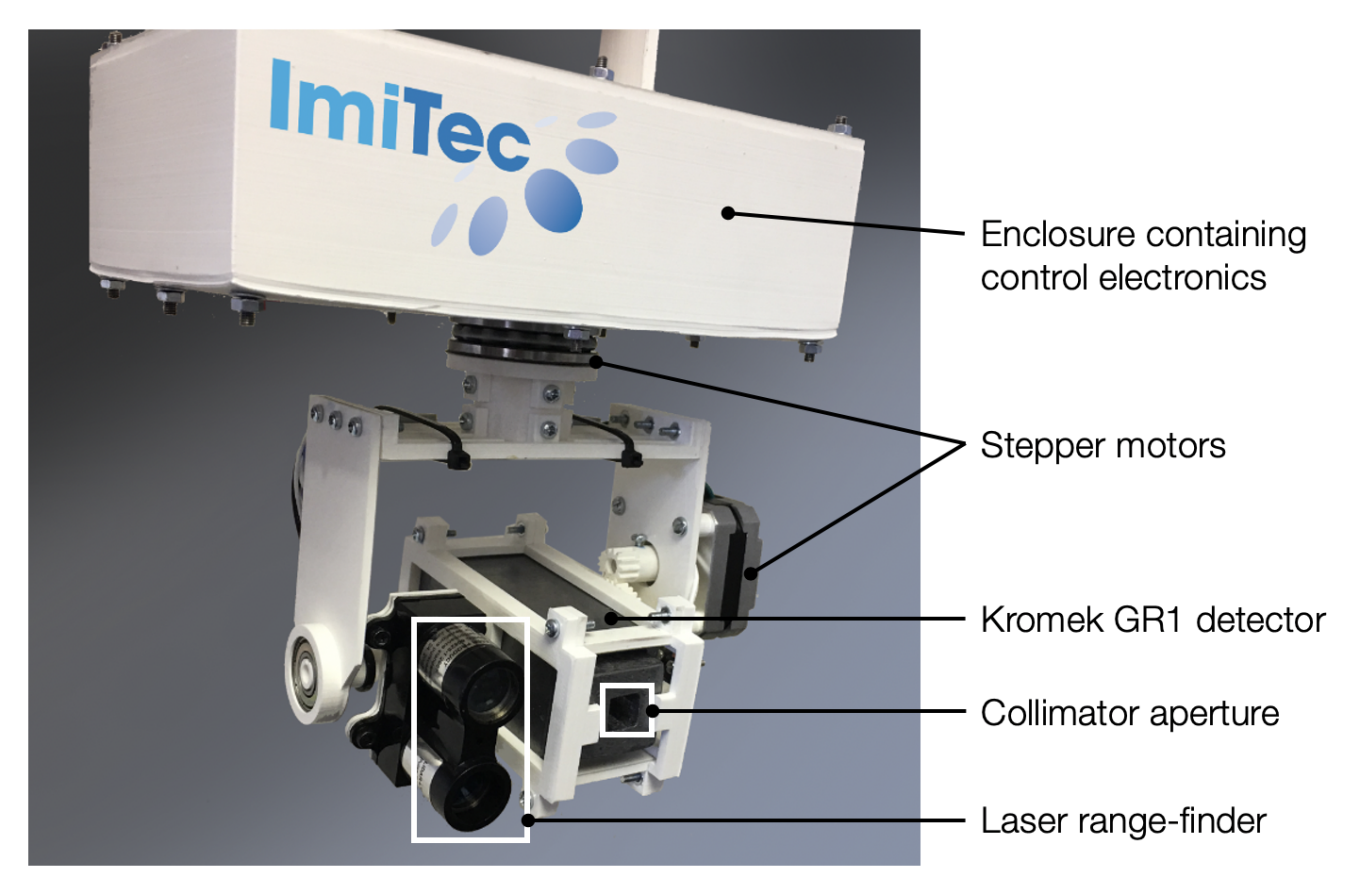
CC-RIAS prototype with components identified

**Figure 2:**
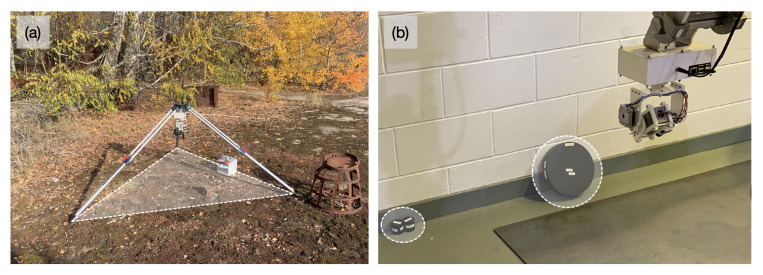
The CC-RIAS device deployed; (**a**) in the field using a tripod to survey for fine-scale fallout particle material, and (**b**) on the end of a PaR manipulation system as used for remote-handling applications at nuclear facilities. The scanning area is highlighted in (**a**), with the ${}^{137}$Cs radioactive sources highlighted in (**b**).

The ‘CC-RIAS’ scanning system developed is shown in Figure 1. The entire platform is 15 cm in height, 15 cm in width and 10 cm in depth; and can be used with either the scanning electronics directed downwards (as shown in Figure 1) or with the detectors on top and the associated control electronics beneath—a deployment mechanism shown in Figure 2a, utilising a tripod to deploy the platform. Such versatility, combined with small dimensions, permits for the radiation scanning system to be deployed through standard 6-inch (15 cm) access penetrations typical of nuclear sites in the UK and worldwide. The detection and mapping element of the CC-RIAS, as identified in Figure 1, comprises a co-incident Kromek GR1 Cadmium Zinc Telluride (CZT) semiconductor-type radiation detector [12,13] and a ‘LiDAR Lite’ single-point laser range-finder. As one of the smallest commercially available solid-state radiation detectors, weighing only 60 g, the GR1 is a room temperature gamma-ray spectrometer with dimensions of 25 mm × 25 mm × 63 mm (active crystal volume 1 cm${}^{3}$), a (gamma) detection range of 30 keV to 3 MeV and an energy resolution of <2.5% Full Width at Half Maximum (FWHM) at 662 keV. To improve directionality onto the detector necessary for the purposes of area scanning and source localisation, it is encased within a 1 cm thick lead collimator with a single square penetration (aperture) of 5 mm × 5 mm at its front—as shown in Figure 1. For deployments where considerable levels of gamma-ray ‘shine’ from multiple sources and reflections/scattering from surfaces was anticipated, a piece of Bi-based ‘Gamma Clay’ was secured to the rear penetration of the collimator/detector to prevent spurious radiation measurements from being recorded that are not associated with the aperture on the front of the detector.

Adjacent to the shielded GR1 on the CC-RIAS detection head, is the single-point (LiDAR) laser range-finder. The Lidar-Lite device is used to obtain rapid distance measurements to the centre line of the detector where the radiation measurement was simultaneously obtained—weighing 22 g with dimensions of 20 mm × 48 mm × 40 mm. This commercially available ‘off-the-shelf’ (COTS) device operates at a wavelength of 904 nm with an 8 mRadian beam divergence and is classified as Class 1, eye safe, during operation. It is capable of distance measurements up to 40 m with an accuracy over the range of ±2 mm.

**Figure 3:**
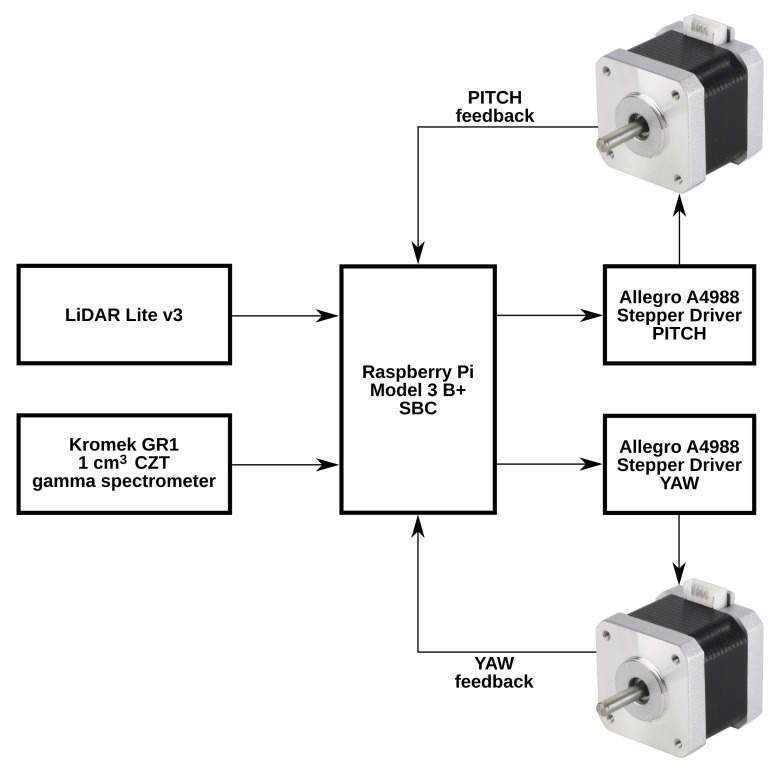
Block diagram of the CC-RIAS prototype.

To move the two coincident detectors, both are mounted on a custom cradle to allow for raster-scanning of a specified, predefined region. Through the use of stepper motors combined with micro-switches, the dual detector setup can pan through a full 360${}^{\circ }$ of rotation and tilt through −40${}^{\circ }$ to 90${}^{\circ }$ (the horizontal plane being at 0${}^{\circ }$). This version of the CC-RIAS platform utilises stepper-motors with a minimum per-step angle of 0.9${}^{\circ }$. This is sufficient, because as modeled below, this corresponds to a focal length of 0.95 m between aperture and detector surface. Control of the stepper motors motion and speed (dwell-time per point) was provided by a Raspberry Pi (Model B) contained within the sealed plastic enclosure, which additionally served to record this positional data alongside measurement values derived from both the gamma-ray spectrometer and the single-point laser range-finder. The resulting PCD is stored on-board of the device or can be retrieved through a wireless connection in real time. The block diagram is illustrated in Figure 3.

The power requirements of the entire system are sufficiently low such that it does not require mains (230 V) power, but can function from a suitable battery source (e.g., lithium polymer or lead-acid battery) to facilitate in-field operations and ‘on-plant’ deployments where conventional power is not available or appropriate. To maximize deployment flexibility, CC-RIAS is equipped with on-board power electronics including a wide input range voltage regulator, which allows CC-RIAS to be powered from any low voltage DC source of 8 V or above, including mobile robotics platforms such as Boston Dynamics’ SPOT walking quadruped. The average power rating depends on the scan interval, and varies between 6 W–8 W. When paired to a standard sealed lead-acid battery with a capacity of 7.5 Ah, the autonomy of the system is ca. 8 h at a battery DoD of 50 %.

Integration time is a critical parameter that is defined as the time the spectrometer aperture is opened while pointed at a given (pitch, yaw) angle set. The Kromek GR1 collects events during this time span, discriminating energy levels with a 12-bit accuracy for a total of 4096 energy bins, and stores them in a memory buffer. A software option enables automatic real-time re-binning to wider energy bins if desired. The integration time *T* is locked and constant for the duration of a scan. The time required for a raster scan is given by:

(1) $$t_{scan}=K_{i}+\sum_{\theta =\theta_{0}}^{\theta_{1}}\left[m_{\theta }+\sum_{\phi =\phi_{0}}^{\phi_{1}}\left(T+m_{\phi }\right)\right]$$

With $t_{scan}$ the total scan duration, *m* the impulse response of, respectively, stepper motors moving for resp. ϕ (yaw) and θ (pitch) adjustment. The time required to initialise and calibrate the system, $K_{i}$, is independent of the scan range or integration time. The stepper motors of type NEMA 17 have an angular resolution of 1.8${}^{\circ }$ in full wave drive, with 16 point micro-stepping supported by the A4988 Allegro stepper drivers. These were chosen for their excellent control stability in comparison to their Trinamic counterpart [14]. Both pitch and yaw have a spur gear drive, corresponding to a minimum step angle of

(2) $$\frac{1.8^{\circ }}{16\cdot N_{\theta }}=\Delta \theta_{min}$$

and

(3) $$\frac{1.8^{\circ }}{16\cdot N_{\phi }}=\Delta \phi_{min}$$

For pitch and yaw, respectively. In the CC-RIAS prototype, $N_{\phi }=N_{\theta }=2$, corresponding to a smallest possible step size of 0.9${}^{\circ }$ in full wave drive mode, and 0.056${}^{\circ }$ in micro-stepping mode. However, the prototype does not feature rotational encoders for positional feedback, increasing $m_{\theta }$ resp. $m_{\phi }$ when operating in open loop. The implemented modes are restricted to either full wave drive with a 0.9${}^{\circ }$ minimum step angle or a 2 point micro-stepping mode with a minimum step angle of 0.45${}^{\circ }$.

When choosing the integration time *T* before starting the scan, care must be taken to not saturate the spectrometer, which happens beyond the threshold of 30 kcps. Optimal *T* values depend on distance to the source(s) and their activity levels, as well as the activity range and desired contrast in the output data. For NORM sources with an activity level below 10 MBq, an integration time *T* = 1–5 s yields the best results at a distance of 1–2 m. For a full range 360${}^{\circ }$ pan scan with tilt angles −40${}^{\circ }$ to 90${}^{\circ }$ and a pixel overlap of 75 % (angular step size 8.75${}^{\circ }$), the total scan time varies between 107 min and 535 min (ca. 9 h).

## 3. Gamma Detector Modeling and Sensitivity

**Figure 4:**
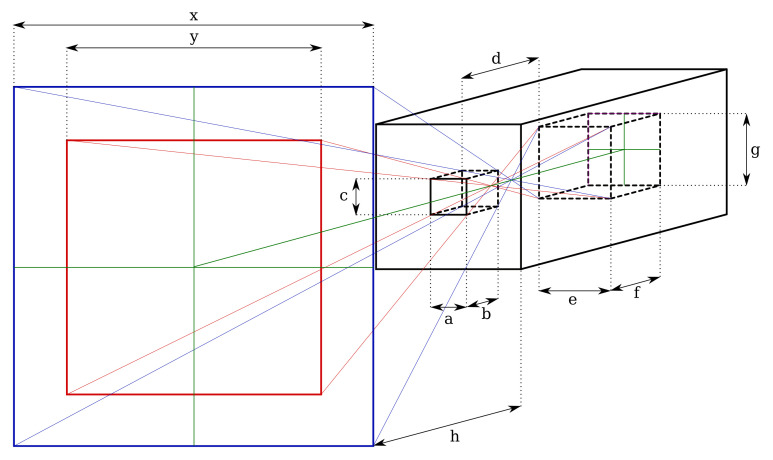
Schematic representation of the detector, showing its CZT detection crystal (cube in the far back with dimensions *e* × *f* × *g*), collimator with aperture dimensions *a* × *c* and thickness *b*, focal length *d*, and pixel projection on a surface at a distance *h*. The green cross represents the reference planes, with the unattenuated pixel area marked in red, and extended (but partially attenuated by the collimator) pixel area is marked in blue.

The detector and its collimator are modeled as shown in Figure 4. The CZT crystal itself is modeled as a prismatic shape with rib lengths *e*, *f*, and *g*, which for the specific case of the Kromek GR1 are of identical length e=f=g=10 mm. The entire crystal is encased in a solid lead collimator with a thickness *b*, and a pin-hole aperture with a rectangular shape (a,c). In the current implementation, this was a=c=5 mm. The distance between the interior of the collimator and the front of the crystal is *d*.

**Figure 5:**
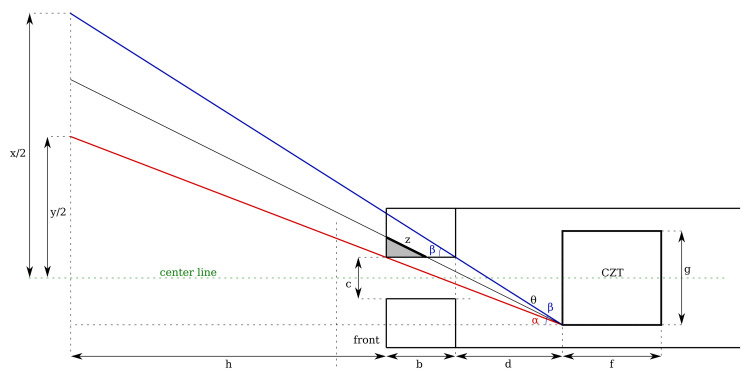
Calculation of the travel distance *z* through solid collimator material for incident angles larger than a direct projection through the collimator aperture allows. Colors are matched with Figure 4.

When capturing a single-point projection at a distance *h* from the aperture, it has a projection with the same shape as the aperture and an area depending on the aperture dimensions, as well as distances *h* and *d*. The projection width/height *y* for a square aperture at distance *h* follows from Figure 5.

(4) $$\tan\alpha =\frac{\frac{g}{2}+\frac{c}{2}}{d+b}=\frac{t}{h}$$

where the section *t* is the projection due to beam divergence beyond the dimensions of the aperture itself, and α the angle between acute angle and center line, as shown in Figure 5. The total area follows as

(5) $$S={\left(\frac{h(g+c)}{d+b}+c\right)}^{2}$$

The first term dominates the equation because the distance to the projection surface is usually much larger than the dimensions of the detector itself. The divergence factor is negatively influenced by the projection surface of the crystal and the size of the aperture, but can be improved by increasing *b* and/or *d*, respectively. If the distance between the aperture and the CZT crystal were increased to infinity, the projection surface becomes

(6) $$\lim_{d\to \infty }{\left(\frac{h(g+c)}{d+b}+c\right)}^{2}=c^{2}$$

which equals the area of the aperture itself assuming a square aperture as in the presented prototype in Figure 1. The smallest theoretically achievable pixel size is, as shown in the equation, the size of the pin hole aperture, however this would require a physically very long detector cavity. Dimensions *b* and *d* must be finite to fit through access ports in real-world nuclear facilities, which presents a design trade-off. With the dimensions of the prototype, the Equation (5) gives a projection function for the CC-RIAS

(7) $$S\left(h\right)={\left(0.6h+0.005\right)}^{2}{\;m}^{2}$$

The pixel/projection size for distances are listed in Table 1. At a distance h=0 the pixel size is 25 mm${}^{2}$ as expected from a square aperture with width and height 5 mm, based on Equation (7). Because of the compact design of the gamma spectrometer scan head, the pixel size diverges relatively quickly and increases to 1 m${}^{2}$ at a distance h=1.658 m. At a distance of 5 m, the pixel size increases to 9 m${}^{2}$ (y=3 m, in Figure 4).

The divergence limits the usefulness of the gamma spectrometer assembly to relatively close surfaces, which tends to be the case when scanning highly active cells, the interior of mixers, dissolvers or other industrial equipment, or waste drums and assorted storage containers.

To model the gamma flux measured by the CZT crystal at a given distance, a correction factor needs to be applied to compensate for partial absorption of the collimator at shallow angles, as shown in the cross section diagram in Figure 5. The same color scheme is applied as in the diagram in Figure 4. The largest angle at which a direct line of sight to the edge of the CZT crystal exists is α, denoted in red, which corresponds to a projection length *y*. At progressive angles, partial signal detection is still possible because of the varying degree of absorption in the collimator. At an angle β (denoted in blue), the travel distance through the collimation material is maximum and a (near) complete absorption is assumed. For angles between α and β, partial absorption takes place, shown at an arbitrary angle θ where scattered gamma photons travel through the collimator over a distance *z* and lose intensity in the process. This is a simplified model that does not take scattering of photons in the collimator into account. It also does not consider Compton scattered gammas reaching the detector through the collimator, which would be perceived as lower energy photons.

The total gamma flux Φ measured by the CZT crystal is the sum of any unattenuated signals originating from an area $y^{2}$ as previously demonstrated (where 0≤θ≤α, and partially attenuated signals for α<θ≤β. As the angle increases, so does the travel distance *z* and proportional attenuation. The travel distance z(θ) can be approximated as;

(8) $$z\left(\theta \right)=\frac{\theta -\alpha }{\beta -\alpha }\cdot \frac{b}{\cos\beta }$$

For any angle θ with α<θ≤β, the projected area has a rib length *v* which equals;

(9) vs.=2(h+b+d)tanθ

The gamma photon attenuation through a collimator is modeled as;

(10) $$\Phi =\Phi_{0}e^{-\mu z}$$

where $Phi_{0}$ is the incident beam intensity, Phi the transmitted radiation intensity, *z* is the thickness of the collimator and μ is the linear attenuation coefficient of the collimator material. μ is a function of the gamma radiation energy and density of the collimator material [15]. If the collimator is relatively thick, and causes scattering of diverging photons, Equation (10) is no longer valid and a so-called build-up factor must be added as a correction:

(11) $$\Phi =\Phi_{0}B\left(E,z\right)e^{-\mu z}$$

The build-up factor B(E,z) is defined as the ratio between the scattered signal fraction and the total signal [16] (p. 215). Combining Equations (11) with (8), the signal transmission for angles α<θ≤β is

(12) $$\Phi =\int_{\alpha }^{\beta }{\left[(h+b+d)\tan\theta \right]}^{2}\Phi_{0}B\left(E,z\right)e^{-\mu z}d\theta$$

with

(13) $$\begin{array}{c} z\left(\theta \right)=\frac{\theta -\alpha }{\beta -\alpha }\cdot \frac{b}{\cos\beta }\\ \alpha =\tan^{-1}\frac{y+g}{2(h+b+d)}\\ \beta =\tan^{-1}\frac{x+g}{2(h+b+d)} \end{array}$$

The total signal intensity projected on the CZT crystal is then;

(14) $$\Phi =\int_{0}^{\alpha }2\left(h+b+d\right)\sin\theta d\theta +\int_{\alpha }^{\beta }{\left[(h+b+d)\sin\theta \right]}^{2}\Phi_{0}B\left(E,z\right)e^{-\mu z}d\theta$$

The first term of the equation corresponds to the unattenuated term $y^{2}\Phi_{0}$, and the second term to the attenuated term where the signal passes through a section of the collimator.

When panning/tilting the gamma camera by an angular increment of ϕ, the angular difference will determine if there is overlap between the current pixel projection and the previous pixel projection. From the square surface model in Equation (5), the width resp. height *R* of a pixel (for a square aperture, as modeled) is

(15) $$R=\sqrt{S(h)}$$

When h>>c, which can be assumed in every practical application, the equation can be simplified to

(16) $$R=\frac{h(g+c)}{d+b}$$

The minimum angle $\phi_{R}$ at which there is no overlap between adjacent pixels for a width/height *R* is

(17) $$\phi_{R}=\sin^{-1}\frac{R}{h}$$

Substituting *R* gives the distance-independent minimum angle $\phi_{R}$:

(18) $$\phi_{R}=\sin^{-1}\frac{g+c}{d+b}$$

The overlap ratio κ between adjacent pixels for any angular increment ϕ can now be modelled as

(19) $$\kappa \left(\phi \right)=\frac{\phi_{R}-\phi }{\phi_{R}}$$

with 0≤κ(ϕ)≤1. For the CC-RIAS prototype, $\phi_{R}\approx 35^{\circ }$.

Data are collected as a 4D PCD with pitch and yaw angles, the distance vector measured by LiDAR, and a 4096 point vector with recorded energies in each of 4096 separate bins in the 30 keV–3 MeV energy range of the GR1. Each sample point in the spherical space is therefore a 4096 point vector. The coordinate system is transformed from a polar to a carthesian system (x,y,z) where (0,0,0) coincides with the centre of the CZT crystal in the spectrometer. Data are saved in a human-readable CSV-format, along with angular step size and integration time as scan parameters.

## 4. Data Collection, Handling, and Processing

To view and process PCD, 3D point cloud processing software *CloudCompare* [17] was chosen because of its ability to handle triangular meshes and calibrated images, thus allowing radiation data to be overlain on top of 3D models of decommissioning environments obtained with photogrammetry. The software has the advantages of being cross platform, which ensures long term support (LTS) for compatibility with CC-RIAS on-board firmware (Debian-based [18]). It is available under a GPL license [17,19].

**Figure 6:**
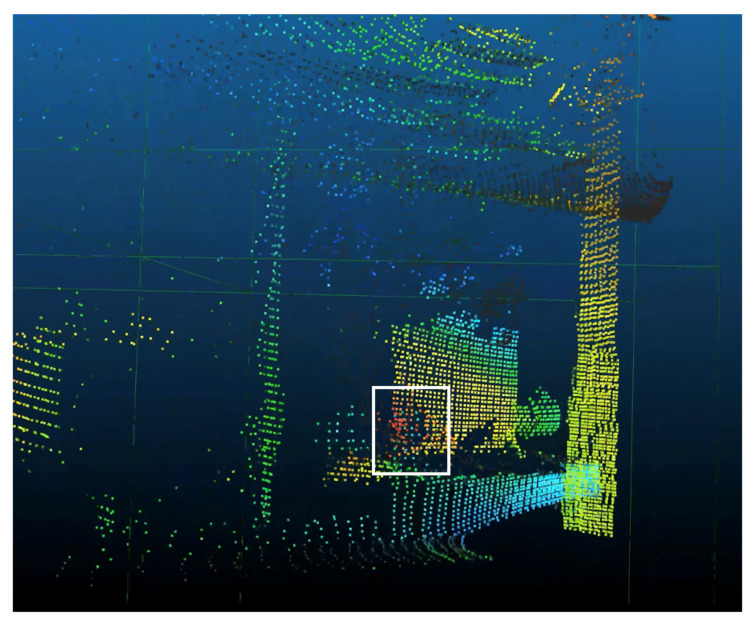
3D radiation intensity (colour-map) point-cloud of an internal space exhibiting vertical faces representative of walls. A region with elevated activity at the centre of the scan is identified—representing an accumulation of radioactive material. Even without visible image overlay, a high density point cloud enables observers to identify object contours with ease. In this scan, the pixel overlap is approx. 700%, causing a spatial averaging effect equivalent to a Gaussian blur. In this scan, the frame projection is ca. 0.8 m tall and 0.6 m wide at a distance of ca. 2.5 m.

The data processing software has 2 modes of operation depending on whether isotopic fingerprinting is desired. In basic mode, the total count rate is calculated as the sum of all counts over the calibrated energy range:

(20) $$cps=\frac{1}{T}\sum_{i=E_{0}}^{4096}E_{i}$$

where $E_{i}$ is the number of counts recorded in each of 4096 bins, cps the number of counts per second, and *T* the scan integration time per measurement point, as defined above. Data in low energy bins are statistically significant but originates from environmental background as well as electronic noise. The distribution is a negative exponential, and even tiny fluctuations in the instantaneous behaviour of the detector heavily influence the counting rate. To keep these counts out of the data set, the low energy bins are discarded with a cut-off at $E_{0}$, with $0<E_{0}<4096$. The value of $E_{0}$ follows from the GR1 calibration process, and remains fixed throughout the operation of the device. When plotted in a 3D space such as CloudCompare, the CPS values are converted to a colour coding of pixels using a Gaussian filter. An example is shown in Figure 6.

In advanced mode, the spectral information is processed using a peak-fitting algorithm the searches for distinctive photo peaks indicative of specific isotopes, or groups of photo peaks. In its current implementation, the algorithm is able to identify monochromatic gamma emissions automatically, i.e., 59 keV for Am-241, 662 keV for Cs-137, etc. A list of identified isotopes is produced as output of a scan along with relative normalised gamma flux levels. Dose rate calculations at the inspected surface using the spectral data have previously been demonstrated [20]. There is currently no visualisation of identified isotopes in an interactive 3D environment such as CloudCompare. All data processing is done after completion of the scan, i.e., post processing rather than real-time processing. It is however possible to stream data from CC-RIAS wirelessly while a scan is in progress, and build up the scene incrementally in near real-time.

## 5. Platform Application and Discussion

Initial deployment and testing of the platform was undertaken at two sites in Ukraine; the Pridneprovsky Chemical Plant (PChP) near Kamianska [21], and Kopachi, a town located within the Chornobyl Exclusion Zone (CEZ) [22]. Both sites pose specific decommissioning challenges, with PChP offering ample indoor environments and the CEZ mainly outdoor environments.

Results obtained during field deployments in Kamianska and Kopachi have gained valuable insights in the operation of the CC-RIAS prototype and its operation modes in different environments. The user can set up the device parameters, monitor the scan progress, and retrieve data remotely through a wireless link with the CC-RIAS. This feature enables the online adjustment and optimization of scan parameters, of which integration time and angular resolution in pitch and yaw are the most important. These parameters depend on the activity levels of hotspots in a given environment, the overall background activity levels, as well as distance to the target(s). For optimal results, CC-RIAS therefore requires its scan parameters to be tweaked for every deployment. Incorrect configuration may result in inadequate event discrimination, saturated detector readings, or excessively long scan intervals.

**Figure 7:**
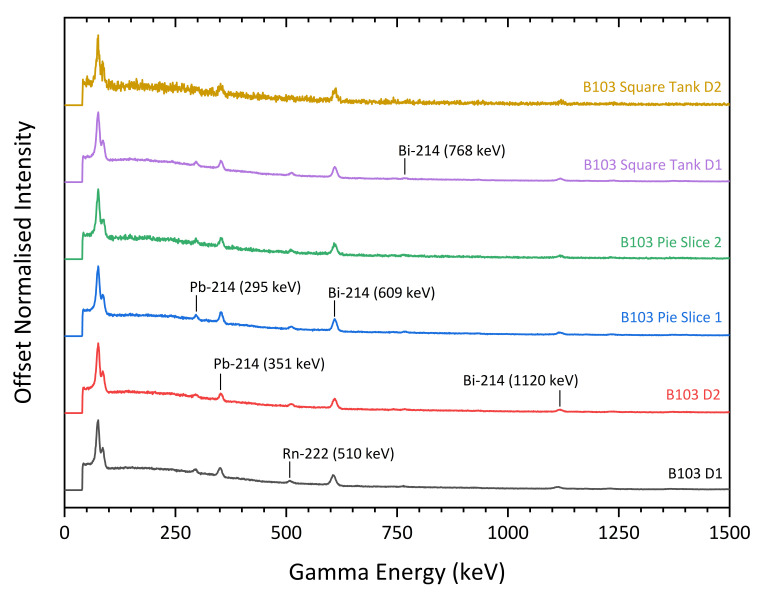
Comparison of gamma spectra captured by the CC-RIAS at different deployment locations within the REE ore processing facilities of PChP. Photopeaks at different energies correspond to decay products of NORM (U and Th series), in varying concentrations as isotopes are selectively removed in the REE purification process. Count rates were normalised to facilitate comparison of spectral recordings at different locations. All graphs are on a linear scale.

**Figure 8:**
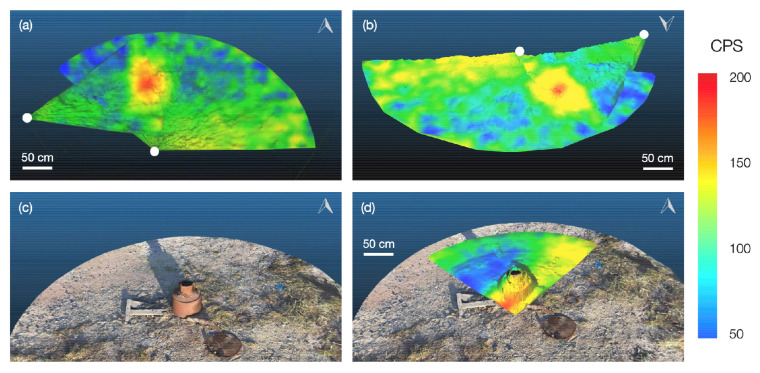
(**a**) and (**b**) overlain radiation intensity scans (CPS) obtained from two perspectives by the CC-RIAS system (position of scanning device indicated by white dots), (**c**) 3-dimensional photogrammetry reconstruction of an object of interest, and (**d**) the 3D photo-realistic rendering overlain with the point-cloud data captured by the CC-RIAS platform.

Examples of data sets obtained during field deployments are shown in Figure 6, Figure 7 and Figure 8. Figure 6 shows a visualisation of the raw PCD, colour coded for radiation intensity (with blue the lowest radiation levels and red the highest radiation levels). The scan subject is a decommissioned REE ore processing facility on the PChP site in Kamianska, with some residual activity remaining in vessels and pipework. Distance to the surface varied between ca. 1.5 m and 5 m. Without additional post processing, it is straight-forward to correlate the PCD with the indoor environment and pinpoint the origin of radiation sources. Walls and ceiling can easily be distinguished, and local hotspots identified. The scan results enable prioritisation of further post operation clean out (POCO) activities. The total integration time for this single 360${}^{\circ }$ scan was ca. 12 h due to the low radiation levels (<500 μSv/h max., integration time *T* = 10 s). Typical count rates in the order of 100–2500 cps.

The gamma-ray spectra obtained from the summation of the spectral results derived from deployments in the PChP test sites are shown in Figure 7. As expected for a site formerly processing U, Th and REE minerals, the signatures resulting from all of the scans is consistent with that of naturally occurring radioactive material (NORM).

**Figure 9:**
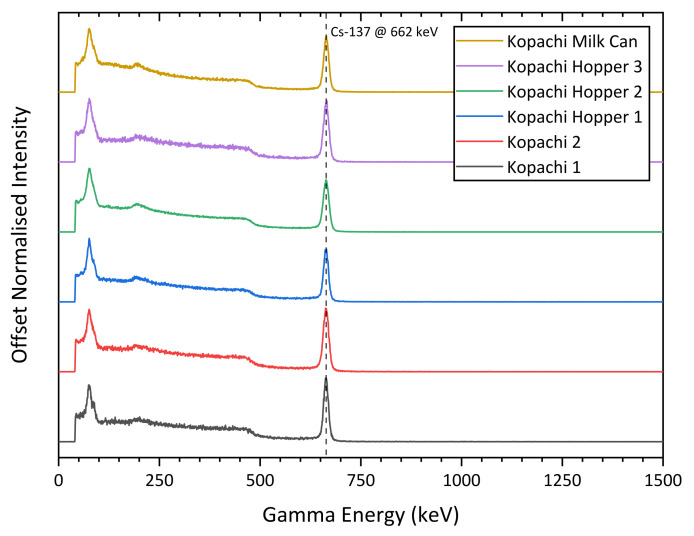
Comparison of gamma spectra captured by the CC-RIAS for different deployment locations in the CEZ, showing consistency in contamination across the exclusion zone. The photopeak at 662 keV identifies ${}^{137}$Cs as it decays into ${}^{137}$Ba from its metastable isomer ${}^{137m}$Ba. CC-RIAS confirms ${}^{137}$Cs is the only gamma-emitting fallout isotope of significance remaining in the CEZ as of 2019. All measurements made at a distance of 1–10 cm from the surface, with normalised peak heights to facilitate comparison.

Figure 8 shows augmented PCD sets captured in outdoor environments in the CEZ. The village of Kopachi within the CEZ, 4.8 km from Unit 4 that underwent catastrophic failure on 26th April 1986, has areas heavily contaminated with particulate radioactive debris originating from the destroyed reactor core. These particles with a size of less than 1 mm have a very high specific activity, with surface dose rates in excess of 10 mSv/h. Locating these particles is especially difficult in the increasingly forested areas around Kopachi, obstructing traditional decontamination efforts. As shown in Figure 8a,b, CC-RIAS is able to isolate the location of particulate hotspots, simplifying their removal and decreasing dose rates for cleanup workers on the ground. CC-RIAS has also proven effective for the detection of radiation sources on the surface or internal of suspected containers such as in Figure 8c,d, where a CC-RIAS captured PCD with radiation data was overlain over a realistic 3D model built with photogrammetry techniques. In both cases, CC-RIAS supplied data that helped to accelerate decontamination and cleanup operations on the ground in the CEZ. Figure 9 illustrates captured gamma spectra at different locations in the CEZ, showing consistent contamination by the radioisotope ${}^{137}$Cs, a dominant constituent of fallout.

## 6. Mobile Deployments

**Figure 10:**
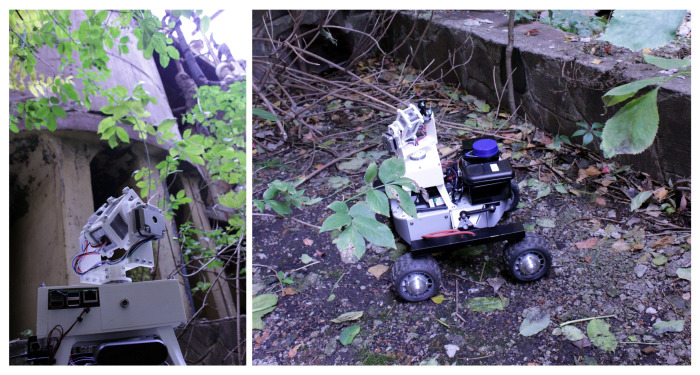
Mobile deployment of CC-RIAS in outdoor environments on the PChP nuclear site in Kamianska, Ukraine. CC-RIAS was deployed to scan waste water tanks to determine radiological contamination.

Figure 2 and Figure 8 illustrate scan setup and results for stationary deployments, either on a tripod (Figure 2a) or on a robotic manipulator in active industrial environments (Figure 2b). However, because of its compact form factor and reduced weight compared to other solutions described in [9], along with its ability to make 360${}^{\circ }$ scans with an angular inclination from −40${}^{\circ }$ to 90${}^{\circ }$, CC-RIAS is also well suited for mobile deployments [23]. To demonstrate this capability, CC-RIAS was mounted on a wheeled rover equipped with a ROS framework for Simultaneous Localisation and Mapping (SLAM). The concept was previously proposed by Sato et al. [11] (Figure 3). In this approach, the LiDAR data is both used for generating a PCD with radiation data in a 3D space, as well as providing position data to the SLAM algorithm. Figure 10 shows deployment of CC-RIAS on top of a wheeled Turtle rover [24], autonomously scanning storage tanks with radiologically contaminated waste on the PChP site in Kamianska, Ukraine.

## 7. Nuclear Decommissioning: Preparation and Deployment

Every nuclear decommissioning environment poses a unique challenge to radiological characterisation, in particular when little or no information on radiological contamination is available. This is often the case for legacy nuclear sites, such as those found around abandoned uranium mines or processing facilities in former Soviet Union republics. In countries with active or historic nuclear weapons programs, such as the UK and USA, the combination of rushed construction of processing facilities and high levels of secrecy have resulted in radiologically contaminated infrastructure that lacks adequate documentation. Many of these facilities have been mothballed for decades and their former staff retired. When preparing for dismantling, engineers are confronted with aging infrastructure, often substantially different from what was originally documented—if such documentation at all survived. Sending radiation experts into these facilities for surveying is irresponsible because the potential radiation hazards (as well as chemical hazards) are unknown. Not only could radiation levels be unexpectedly high, facilities with fissile material also pose a criticality risk because water-rich human bodies act as neutron moderators. CC-RIAS is designed to be at the front line of these unknown cell characterisation challenges. Facilities that have been long abandoned are often found to be no longer accessible through their original access points: steel doors are rusted shut or passage ways have been closed with brick walls or concrete to keep contamination inside. If storage tanks are present that may have disintagrated or leaked, the floors of radiologically contaminated cells can be flooded, and opening shut entrances is therefore a hazardous undertaking.

Because of the compact design, CC-RIAS can be deployed through access ports of only 20 cm diameter. These ports can either be already present in the infrastructure, or can be drilled in walls and ceilings for the explicit purpose of CC-RIAS deployment. When deployed on an aluminium rod or similar gantry system through circular ports, operators can easily stay out of direct shine paths. When pre-configured for a quick scan, integration time is typically 1 s and angular step size 10${}^{\circ }$ over a range of 180${}^{\circ }$. This enables the acquisition of a point cloud as shown in Figure 6 which highlights localised hotspots. As discussed previously, the spectral data is then used to calculate a dose map approximating surface dose rates on surfaces within line of sight of CC-RIAS [20]. Using the inverse square law and inversion algorithms, the dose rate at different locations (within line of sight) of CC-RIAS can be approximated in a 3D environment. The more surfaces are in view, the close the approximation will approach actual values. Obscured (unseen) surfaces present a risk since they can contribute a dose to a point in a 3D space, leading CC-RIAS to underestimate the actual dose rates. Based on these dose map estimates, further decommissioning efforts can be planned because it allows preliminary radiation hazard assessment. This means, for example, whether it is warranted for a human to venture inside the cell and deploy CC-RIAS at a distance further from the access point, or whether it should be deployed on a mobile robotic platform instead. Subsequent scans reveal obscured areas and incrementally complete the point cloud by contributing previously unseen sections of the cell, improving accuracy of dose maps. Spectral data simultaneously provides data on the nature of the radiological contamination, for example ${}^{241}$Am can be detected by its 59.5 keV gamma photopeak and is usually an indicator of the presence of ${}^{241}$Pu (${}^{241}$Pu decays into ${}^{241}$Am through beta decay). Fissionable with fast neutrons, ${}^{241}$Am may indicate the presence of a potential criticality risk when moving materials during the dismantling process of installations.

After coarse scans, when areas of interest have been identified as in Figure 6, more detailed scans can be performed by “zooming” to smaller areas: increasing integration time and decreasing angular step size. As such, particularly in high dose environments exceeding 10 mSv/h, CC-RIAS can be a useful tool to aid in the characterisation process of unknown legacy cells.

## 8. Summary and Conclusions

The gamma radiation scanning system presented in this work was designed for characterization of unknown environments and fingerprinting radioactive contamination. Intended primarily for nuclear decommissioning and waste characterisation applications, the objectives to keep the design as compact as possible and disposable if necessary, were achieved. In its current envelope, the CC-RIAS prototype is small enough to fit through 15 cm access ports in nuclear facilities. A commercial CZT-based gamma spectrometer unit encased in a lead colimator was chosen to keep costs to a minimum. The consequences for the resolution and performance of the device were theoretically modeled. It has an energy range from 30 keV to max. 3 MeV, saturation point of 30 kcps, maximum useful range of 5 m, and view angle of 35${}^{\circ }$ (adjustable).

Secondly, two case studies of deployments of the CC-RIAS prototype in Ukraine were discussed. In the first case study, a legacy Soviet era nuclear facility, the CC-RIAS prototype was demonstrated in an interior environment where industrial apparatus for chemical separation of radioactive isotopes was characterized. The presented results demonstrate that a 3D model of the facilities interior could be built, remotely, within 24 h, that allows accurate localization of radioactive compounds and contamination. The collected CC-RIAS data has proven to be sufficiently accurate to prioritize decommissioning efforts in this case study, its primary objective.

As a second case study, CC-RIAS was deployed in an exterior environment near the town of Kopachi in the CEZ to identify locations of highly active particles and contamination of a concrete surface. The measurements were made in an inverted CC-RIAS configuration, and the locations of individual hot-spots could easily be pinpointed on the point cloud, enabling the successful retrieval and disposal of these highly active particles.

## Figures and Tables

**Table 1:** Projection surface size in function of the distance from the aperture.

Distance *h* (m)	Surface *S* (m${}^{2}$)
0	25 × $10^{-6}$
0.5	0.093
1	0.366
1.5	0.819
1.658	1
2	1.452
2.5	2.265
3	3.258
3.5	4.431
4	5.784
4.5	7.317
5	9.030

## Data Availability

All point cloud data (PCD) and spectral data used to generate the graphs and renderings in this paper are available under Creative Commons 4.0 license (CC BY-NC-SA 4.0) at doi:10.13140/RG.2.2.28932.65920.

## Abbreviations

The following abbreviations are used in this manuscript:

SiPM	Silicon Photomultiplier
PMT	Photomultiplier Tube
PCD	Point Cloud Data
VIS	Visible light
LiDAR	Light Detection Furthermore, Ranging
FoV	Field of View
CC-RIAS	Cell Characterisation Remote Isotopic Analysis System
PTU	Pan-Tilt Unit
UK	United Kingdom
CZT	Cadmium Zinc Telluride
FWHM	Full Width at Half Maximum
COTS	Commercially available ’off-the-shelf’
RPi	Raspberry Pi
LTS	Long Term Support
PChP	Pridneprovsky Chemical Plant
CEZ	Chornobyl Exclusion Zone
REE	Rare Earth Element
NORM	Naturally Occurring Radioactive Materials
POCO	Post Operations Clean Out
ROS	Robotics Operating System
SLAM	Simultaneous Localisation and Mapping
NCNR	National Centre for Nuclear Robotics
RAIN	Robotics and AI in Nuclear
DoD	Depth of discharge
